# Kinetic study of glyphosate biodegradation by actinobacterial consortium RH1: implications for bioremediation

**DOI:** 10.3389/fbioe.2026.1820249

**Published:** 2026-07-28

**Authors:** Hadjer Rebai, Rym Salah‐Tazdaït, Djaber Tazdaït, Essam Nageh Sholkamy, Mohamed A. A. Abdelhamid, Seung Pil Pack, Ashraf Aly Hassan, Prakasam Thanka Pratheesh, Hazim O. Khalifa, Allaoueddine Boudemagh

**Affiliations:** 1 Department of Microbiology, Constantine 1- Frères Mentouri University, Constantine, Algeria; 2 Bioengineering and Process Engineering Laboratory (BIOGEP), École Nationale Polytechnique, Algiers, Algeria; 3 Department of Nature and Life Sciences, Faculty of Sciences, University of Algiers, Benyoucef Benkhedda, Algiers, Algeria; 4 Department of Botany and Microbiology, College of Science, King Saud University, Riyadh, Saudi Arabia; 5 Biology Department, Faculty of Education and Arts, Sohar University, Sohar, Oman; 6 Department of Botany and Microbiology, Faculty of Science, Minia University, Minia, Egypt; 7 Department of Biotechnology and Bioinformatics, Korea University, Sejong, Republic of Korea; 8 Department of Civil and Environmental Engineering, United Arab Emirates University, Al Ain, United Arab Emirates; 9 Department of Veterinary Medicine, College of Agriculture and Veterinary Medicine, United Arab Emirates University, Al Ain, United Arab Emirates; 10 United Arab Emirates University (UAEU) Center for Public Policy and Leadership, United Arab Emirates University, Al Ain, United Arab Emirates; 11 Laboratory of Molecular and Cellular Biology, Constantine 1- Frères Mentouri University, Constantine, Algeria

**Keywords:** actinobacteria, agricultural soil, biodegradation, glyphosate, herbicide, kinetic models

## Abstract

**Background:**

The excessive use of glyphosate herbicide in agriculture adversely affects the environment and soil health. Bioremediation using microbial consortia offers an efficient approach for transforming pesticides into less harmful products.

**Objectives:**

The study investigated glyphosate biodegradation by the RH1 consortium and identified suitable kinetic models to support bioremediation.

**Methods:**

Glyphosate biodegradation was investigated at concentrations of 1–200 mg/L using the RH1 microbial consortium comprising four *Streptomyces* strains (SPA2, IT, Herb, and SC). These strains had been previously isolated and validated for their individual glyphosate-degrading capacity. Consortium activity was assessed under optimized conditions (30 °C, pH 7.2, and 4% inoculum). Degradation kinetics were modeled using several established approaches, including Haldane–Andrews, Yano and Koga, Tseng and Wayman, and Webb. Comparative functional analysis was further performed at 50 mg/L using total organic carbon (TOC) quantification and ATR-FTIR spectroscopy to distinguish consortium performance relative to single-strain treatments.

**Results:**

Following 15 days of incubation under optimized conditions, the RH1 consortium achieved high glyphosate removal efficiencies of 92.2%, 87.2%, 91.72%, 92.06%, 54.11%, and 37.085% at initial concentrations of 1, 10, 25, 50, 100, and 200 mg/L, respectively. Notably, at 50 mg/L the consortium demonstrated the highest degradation rate compared with pure-culture treatments. Kinetic evaluation indicated that the Haldane–Andrews model best described the degradation behavior (F = 65.49, P = 0.00074, *R* ^2^ = 0.976). At the same concentration (50 mg/L), total organic carbon (TOC) decreased by 91.03%, corroborating substantial mineralization or conversion of organic constituents. ATR-FTIR spectroscopy further confirmed glyphosate transformation by showing alterations in the pesticide’s chemical bonding patterns after biodegradation, consistent with structural modification of the molecule.

**Conclusion:**

The RH1 actinobacterial consortium efficiently degraded glyphosate, fitting best to the Haldane–Andrews kinetic model. Significant TOC reduction and ATR-FTIR–confirmed structural changes indicate effective glyphosate transformation, highlighting RH1’s potential for bioremediation of glyphosate-contaminated soils.

## 1 Introduction

Pesticides are plant protection products used worldwide in agriculture to improve the quality and quantity of agricultural products and thus achieve food security. However, they can have a negative impact on the environment when transported by land, air, and water, thereby harming the health of living beings (Abdelhamid et al., 2024; Zhou et al., 2024).

Bioremediation represents a promising and effective approach for environmental cleanup. Numerous studies have shown that microorganisms can biodegrade pesticides through metabolic activity and adaptive responses to these contaminants. Nevertheless, prior research has largely emphasized biodegradation using single microbial strains, rather than evaluating consortium-based strategies (Rebai et al., 2024; Rebai et al., 2026; Muthukumaravel et al., 2024; Mansouri et al., 2025; Alatassi et al., 2025). In complex ecosystems, single strains often have limited pesticide-degradation capacity, whereas a bacterial consortium driven by enzymatic and metabolic diversity and enhanced adaptability can more effectively mineralize these pollutants (Rosariastuti et al., 2023; Jokhakar and Dudhagara, 2022). Microbial consortia can generally be obtained from combinations of existing active strains or by domestication from polluted environments (Zhang et al., 2024). Recent research reports have focused on the degradation of pesticides by consortia (Mohanty and Jena, 2022; Dong et al., 2025; Yadav et al., 2025).

Glyphosate (N-phosphonomethyl glycine) is one of the most widely used herbicides in the world, due to its broad-spectrum action against annual and perennial weeds (Zabaloy et al., 2022). The use of glyphosate has boomed globally, with approximately 600,000 to 750,000 tonnes of glyphosate currently being used each year worldwide, and it is estimated that this number will increase from 740,000 to 920,000 tonnes by 2025 (Mohy-Ud-Din et al., 2023).

Glyphosate inhibits the shikimate pathway by blocking the synthesis of EPSP (via competition with phosphoenolpyruvate), thereby preventing the formation of aromatic amino acids (phenylalanine, tryptophan, and tyrosine) required for protein synthesis in plants and leading to plant death within approximately 1–3 weeks after application (Ojelade et al., 2022; Boocock and Coggins, 1983). Glyphosate was first developed in the United States (USA) in 1971 (Mohy-Ud-Din et al., 2023). Glyphosate is widely used because it is relatively non-toxic to animals and humans, which lack the shikimate pathway; however, excessive use has caused adverse environmental impacts and health risks to living organisms (Wang et al., 2016).

There are only a few reports that have studied the biodegradation of glyphosate herbicide by a consortium. Malla et al. (2023) evaluated the ability of a consortium consisting of the bacteria *Lactobacillus plantarum*, *Lactobacillus rhamnosus* and *Bacillus shackletonii* to degrade glyphosate, chlorpyrifos and cypermethrin and found that this consortium degraded 87.01% of glyphosate. Zhang et al., 2024 found that the consortium YS622 efficiently breaks down 100% of the 50 mg/L glyphosate herbicide. Mohanty and Jena (2022) studied the ability of the SMC1 monologue containing *Serratia ureilytica*, *Enterobacter cloacae* and *Pseudomonas putida* to degrade different herbicides, including glyphosate.

In the biodegradation of xenobiotics like pesticides, high concentrations of the target substrates or their resulting metabolites can paradoxically act as inhibitors to the very microorganisms responsible for their degradation (Hanano et al., 2025; Huang et al., 2023). While numerous studies have explored glyphosate biodegradation, there is a significant research gap regarding the herbicide’s auto-inhibitory effects on microbial growth and enzyme activity—a critical factor for optimizing bioremediation that can be addressed through established mathematical inhibition models (Gaonkar et al., 2019; Tazdaït et al., 2018).

This study investigated the ability of a novel actinobacterial consortium (strains SPA2, IT, Herb, and SC) to degrade glyphosate under optimized conditions, utilizing kinetic modelling to evaluate the herbicide’s inhibitory effects on microbial growth and comparing the consortium’s efficiency against individual strains.

## 2 Materials and methods

### 2.1 Bacterial strains

The bacterial strains *Streptomyces* sp. strain SPA2 (accession number pp413753), *Streptomyces rochei*. strain IT (accession number pp413751), *Streptomyces variabilis*. strain Herb (accession number pp413750), and *Streptomyces griseoincarnatus*. strain SC (accession number PP413754) previously isolated and tested for the degradation of glyphosate (Rebai et al., 2025).

### 2.2 Antagonism test

The method of Fuentes et al. (2013) was used to test for the presence of antagonistic effects among the actinobacteria studied. In summary, the strains were spread in ISP2 medium (International *Streptomyces* Project 2) with one strain placed at the center of a petri dish and cross-confronted with the other strains, in all possible combinations. Then, the dishes were incubated at 30 °C for 7 days. The presence of an inhibition of the growth of a strain indicates the presence of an antagonistic effect.

### 2.3 Glyphosate biodegradation by consortium RH1

The herbicide biodegradation was evaluated with the consortium RH1. Single strains were individually pre-cultured in liquid ISP2 medium (30 mL), and then incubated at 30 °C with 200 rpm agitation for 3 days. After centrifugation at 6,000 g for 10 min at room temperature, the pellets were washed and suspended in sterile saline (0.85% NaCl) to set an optical density (OD) of 1.0 at 590 nm (Fuentes et al., 2017; Wahla et al., 2019). Then, 4.0 mg/L of the mixed strains (in a 1:1:1:1 proportion) was added to 250 mL Erlenmeyer flasks containing 100 mL of Mineral salt medium (MSM) with glyphosate at different concentrations (1 mg/L, 10 mg/L, 25 mg/L, 50 mg/L, 100 mg/L, and 200 mg/L) (Wahla et al., 2019) The flasks were incubated at 30 °C under agitation at 200 rpm for 15 days. Samples were taken every 3 days to determine microbial growth by measuring the dry weight and the residual concentration of glyphosate was determined by a colorimetric method based on the reaction of glyphosate with ninhydrin, according to the protocol described by Bhaskara and Nagaraja (2006). The procedure was carried out in triplicate with the respective abiotic controls (pesticides-contaminated MSM without inoculum).

### 2.4 Total organic carbon analysis

The amount of organic carbon reduced after glyphosate biodegradation at a concentration of 50 mg/L was measured using a TOC-L Shimadzu analyzer. After 15 days of incubation, samples were centrifuged, filtered, and diluted before analysis (Korkmaz et al., 2021).

### 2.5 ATR-FTIR analysis of glyphosate

The degradation of the herbicide glyphosate by the consortium RH1 at a concentration of 50 mg/L was monitored using ATR-FTIR spectroscopy (Griffiths, 1983).

### 2.6 Kinetic analysis

#### 2.6.1 Cell growth and glyphosate biodegradation kinetic studies

Specific growth rate [*μ* (1/Day)] values were determined during the exponential growth phase using the following equation (Equation 1):

$$\ln\frac{X}{X_{0}}=\mu \times t$$ (1)

where *X*_*0*_, *X* representing the initial cell concentration (g/L) and cell concentration at *t*, respectively.

The estimation of the specific glyphosate degradation rates (*q*) for each initial glyphosate concentration was performed based on Equation 2 (Kovárová-Kovar and Egli, 1998):

$$q=\frac{\mu }{Y}$$ (2)

where *Y* (g_biomass_/g_glyphosate_) is the growth yield of the bacterial consortium used.

The following equation (Equation 3) has been applied to estimate the value of the minimum concentration of glyphosate (*S*_*min*_ (mg/L)) at which it is degraded (Equation 3):

$$S_{\min\;}=\frac{b\times K_{s}}{\left(Y\times q_{\max}\right)-b}$$ (3)

where *b*, *K*_*s*_ and *q*_*max*_ are the first-order decay coefficient (1/Day) for glyphosate, the half-saturation constant (mg/L), and the maximum specific glyphosate degradation rate (1/Day), respectively.

The parameter *b* was determined by plotting a graph of ln (glyphosate concentration) against time. The slope of the resulting plot is *b*.

On the other hand, *Ks* and *q*_*max*_ (1/Day) were determined based on the Michaelis-Menten equation, which describes substrate biodegradation (Equation 4). This equation relies on *q*, which is directly proportional to the initial substrate concentration when the latter is substantially less than *Ks* (Tazdaït and Salah Tazdaït, 2025).

$$q=q_{\max}\times \frac{S}{K_{s}+S}$$ (4)

The two parameters (*Ks* and *q*_*max*_) were found by employing the Lineweaver-Burk plot (Equation 5):

$$\frac{1}{q}=\left(\frac{K_{s}}{q_{\max}}\right)\times \left(\frac{1}{S}\right)+\left(\frac{1}{q_{\max}}\right)$$ (5)

#### 2.6.2 Model equations used for cell growth

The four inhibition models given below were tested to describe the effect of the initial glyphosate concentration on the growth performance of the bacterial consortium tested in this study. The data predicted by the mathematical models were compared to those obtained experimentally. The models were resolved using the nonlinear regression method via Statistica (release 8.0 software, StatSoft Inc., USA), which employs the nonlinear least squares model estimation based on the Levenberg–Marquardt algorithm to minimize the sum of squared residuals and estimate the kinetic parameters. Statistical significance in the experimental investigation was set to p < 0.05.

The Haldane-Andrews model (Edwards, 1970) (Equation 6) is among the most used models for depicting the inhibitory influence of substrates on microbial proliferation under substrate-limiting conditions (Geed et al., 2022).

$$\mu =\left(\mu_{\max\;}\times S\right)/\;\left(K_{s}+S+\left(S^{2}/\;K_{i}\right)\right)$$ (6)

where *K*_*i*_ is the inhibition constant (mg/L).

Yano and Koga (1969) elaborated a model to describe the inhibitory effect of different substrates in a continuous bioprocess (Equation 7):

$$\mu =\left(\mu_{\max\;}\times S\right)\;/\left(K_{s}+S+\left(\frac{S^{2}}{K_{1}}\right)+\left(\frac{S^{3}}{K_{2}^{2}}\;\right)\right)$$ (7)

where *K*_*1*_ and *K*_*2*_ (mg/L) are positive constants that characterize the substrate inhibitory action.

The model introduced by Tseng and Wayman (1975) (Equation 8) considers the inhibitory impact of varying substrates (ethyl alcohol, 1-butanol, ethyl acetate, and acetic acid) on the growth of two yeast species:

$$\mu =\left(\frac{\mu_{\max\;}\times S}{K_{s}+S}\right)-\left(K_{i}\times \left(S-S_{m}\right)\right)$$ (8)

where *S*_*m*_ (mg/L) is the substrate concentration at which microbial growth stops.

Webb model (Edwards, 1970) (Equation 9) integrates an allosteric action of enzymes with a dimensionless empirical parameter (*β*) to represent the growth inhibition induced by substrates kinetically:

$$\mu =\mu_{\max\;}\times S\times \left(\left(1+\left(\beta \times \frac{S}{K_{i}}\right)\right)/\left(K_{s}+S+\left(\frac{S^{2}}{K_{i}}\right)\right)\right)$$ (9)

Levenspiel revisited the model of Monod and proposed the following model (Levenspiel, 1980) (Equation 10):

$$\mu =\mu_{\max\;}\times \left(1-\frac{S}{K_{i}}\right)\times \left(\frac{S}{K_{s}+S}\;\right)$$ (10)

### 2.7 Statistical analysis

All experiments were performed in triplicate. The results are presented with standard error. Two-way ANOVA and Tukey’s *post hoc* tests were used to establish statistical significance at a threshold of p ≤ 0.05.

## 3 Results

### 3.1 Antagonistic activity test

**FIGURE 1:**
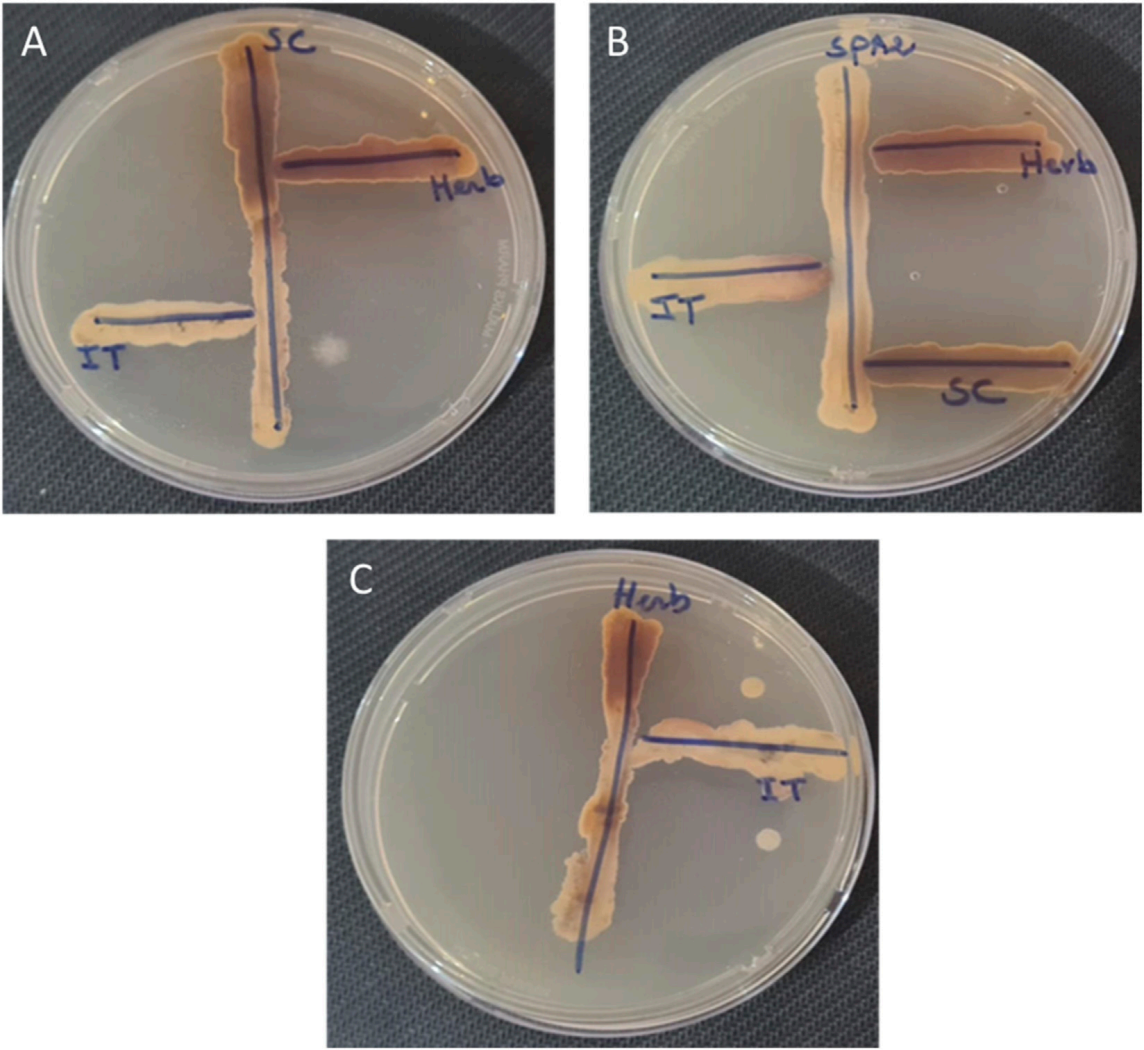
Antagonistic activity test among the target actinobacterial strains. **(A)** SC against Herb and IT; **(B)** SPA2 against Herb, IT, and SC; **(C)** Herb against IT.

The results showed an absence of antagonistic interactions among the tested actinobacteria, which indicates their potential compatibility for co-cultivation as a defined microbial consortium (Figure 1).

### 3.2 Effect of glyphosate concentration on the growth of the RH1 consortium

**FIGURE 2:**
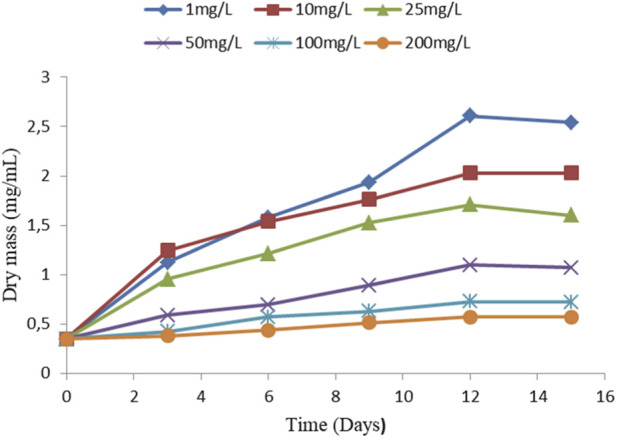
Growth kinetics of the actinobacterial consortium RH1 under optimal conditions with varying initial glyphosate concentrations.

The growth of the consortium RH1 was evaluated every 3 days at different concentrations of glyphosate (from 1 mg/L to 200 mg/L) as the sole source of carbon, and the results are presented in Figure 2.

The actinobacterial consortium showed continuous growth at all tested concentrations during the first 12 days of incubation (exponential phase). However, the growth rate was variable according to the concentration. At low concentrations, from 1 mg/L to 25 mg/L, the consortium showed a rapid growth. A moderate growth was observed at a concentration of 50 mg/L, and at the highest concentrations, 100 mg/L and 200 mg/L, the growth increases slowly. The highest dry mass at all concentrations was observed on day 12 of incubation. Then, it remains stable for the last days of incubation (the stationary phase). Significant differences in growth of consortium RH1 at all tested concentrations (p-value<0.05).

### 3.3 Effect of glyphosate concentrations on its biodegradation by consortium RH1

**FIGURE 3:**
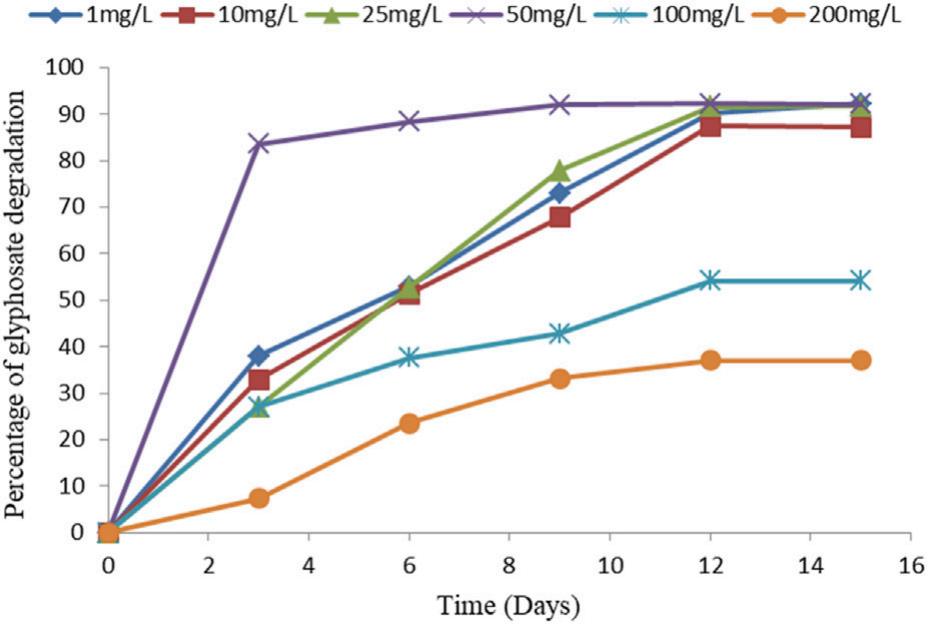
Effect of different initial glyphosate concentrations on rates of degradation by the consortium RH1 after 15 days of incubation.

By adding the herbicide glyphosate as the sole source of carbon at varying concentrations to the medium MSM, the consortium demonstrated the ability to remove glyphosate at all tested concentrations (Figure 3). The highest rate of degradation of the initial concentrations of glyphosate (1 mg/L, 10 mg/L, 25 mg/L, and 50 mg/L) was observed, with percentages of 92.2%, 87.2%, 91.72%, and 92.06%, respectively, within the first 12 days of incubation. Then it remains stable under the optimal condition (temperature of 30 °C, pH 7.2 and inoculum size 4%) tested for the individually incubated strains. At the highest tested concentration, 200 mg/L, a low percentage of biodegradation was observed (37.08%), as well as a moderate percentage (54.11%) at the concentration 100 mg/L.

The consortium RH1 rapidly degraded the herbicide glyphosate within the first 12 days for low concentrations (ranging from 1 mg/L to 50 mg/L). It was moderate at 100 mg/L, and low at 200 mg/L. The highest glyphosate concentrations inhibit its own biodegradation by the consortium RH1. The concentrations of the herbicide showed significant effects on its biodegradation by the consortium (p-value < 0.05).

### 3.4 Total organic carbon analysis

The results of the reduction of total organic carbon by the actinobacteria consortium RH1, using glyphosate as the sole carbon source at a concentration of 50 mg/L, showed that the consortium was able to eliminate 91.03% of the total organic carbon during a 15-day incubation period.

### 3.5 ATR-FTIR analysis

**FIGURE 4:**
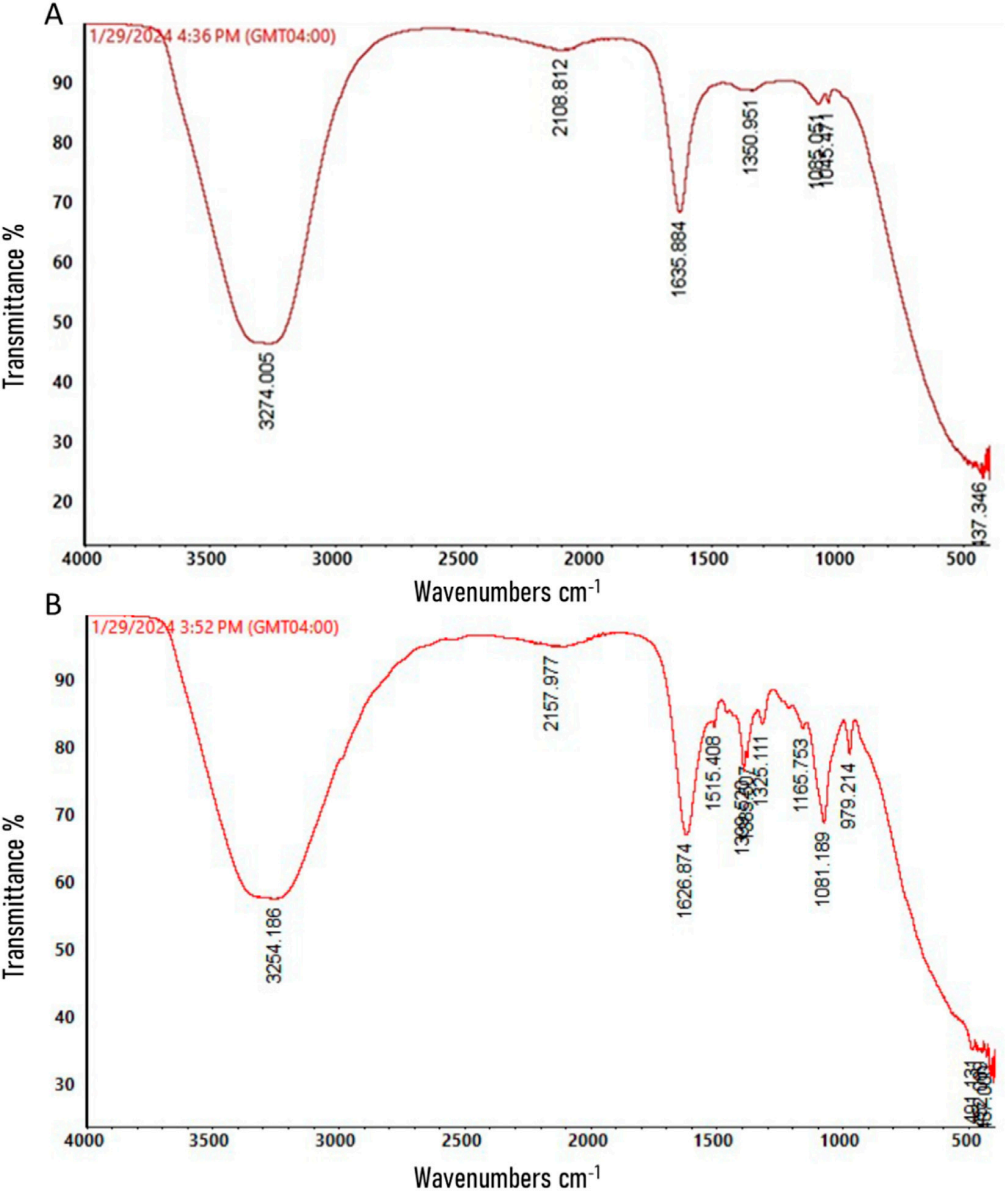
ATR-FTIR analysis of glyphosate herbicide: **(A)** in the consortium RH1; **(B)** in the control.

ATR-FTIR analysis of glyphosate and its biodegraded metabolites is presented in Figure 4. This analysis showed changes in the structure of glyphosate biodegraded by the consortium RH1. A peak located at 1515.408 cm^−1^ is found only in the glyphosate control, and disappears in the consortium, corresponding to the amide group II. The C-O bond peak appeared in the consortium sample at 1045,471 cm^−1^, and it was absent in the control.

### 3.6 Kinetics study

#### 3.6.1 Effect of initial glyphosate concentration on cell growth through a modelling study

**FIGURE 5:**
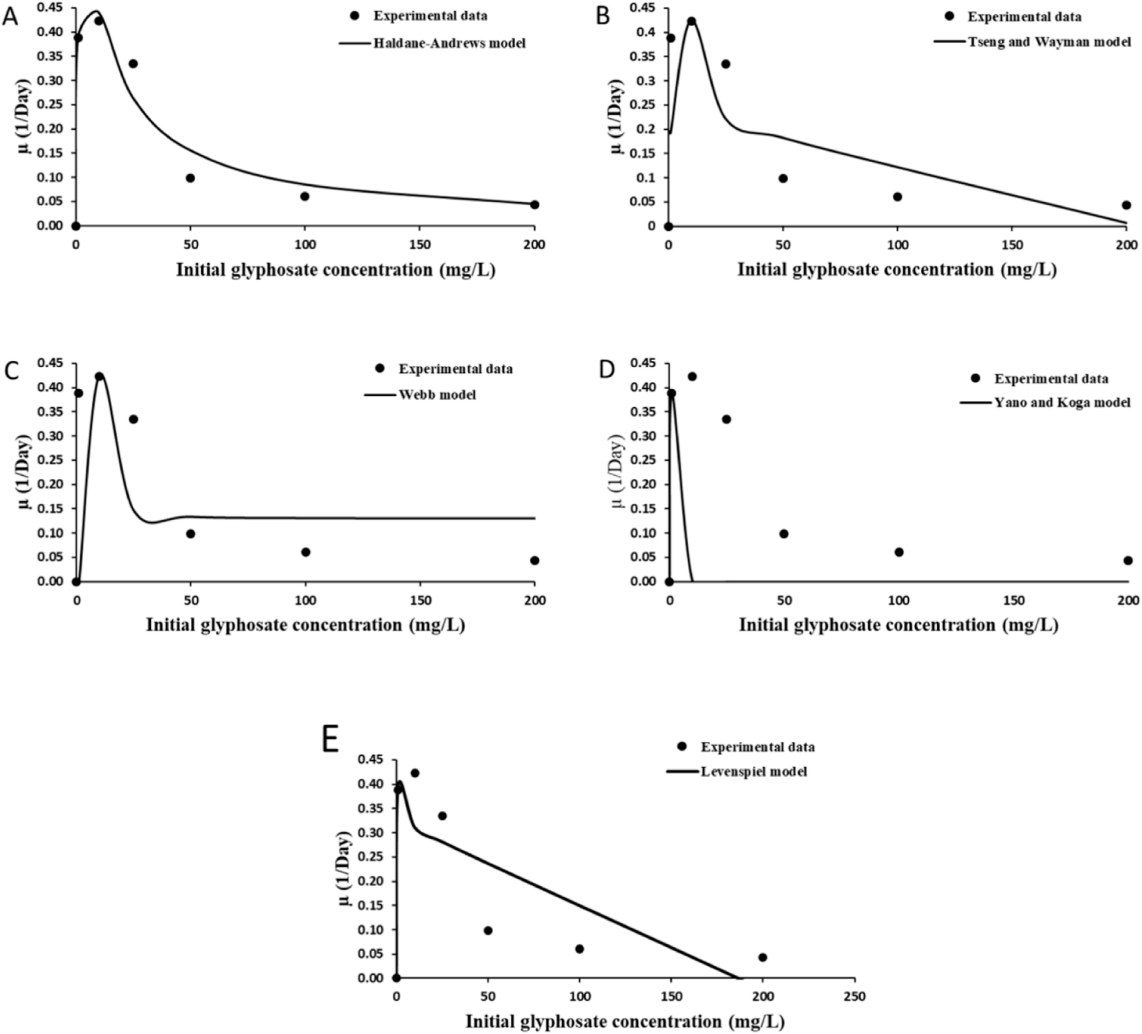
Experimental and predicted specific growth rates of the consortium RH1 at varying glyphosate concentrations, assessed using different mathematical models: **(A)** Andrews-Haldane model; **(B)** Tseng and Wayman model; **(C)** Webb model; **(D)** Yano and Koga model, **(E)** Levenspiel model.

Microbial specific growth rate (*µ*) for the consortium RH1 was calculated at various initial glyphosate concentrations (1–200 mg/L) by plotting ln (*X*/*X*_*0*_) as a function of time and using the straight line of the plot. The variation of µ in relation to the initial concentration of glyphosate was plotted, as depicted in Figure 5. Haldane-Andrews, Yano and Koga, Tseng and Wayman, and Webb kinetic models were tested to fit the experimental data. The estimated statistics parameters (coefficient of correlation (*R* ^*2*^), Fisher (*F*) (ratio of two variances) and *P* (the probability of how much the variation of initial glyphosate concentrations can be used to predict the *µ* values for each mathematical model) values), and the biokinetic parameters of these two models are given in Tables 1, 2, respectively. As can be seen from Figure 5, the growth of the microbial consortium, expressed by µ, increased with the initial glyphosate concentration and attained a threshold (0.42 1/Day) at the concentration of 10 mg/L. Beyond this critical concentration, the growth of the bacteria decreased upon increasing the initial glyphosate concentration. This is very likely due to a microbial inhibitory growth exerted by glyphosate when it surpasses the specific concentration of 10 mg/L. The experimental data best fit the Haldane-Andrews kinetic model, having *F*, *P*, and *R* ^*2*^ values of 65.49, 0.00074, and 0.976, respectively (Table 1), which implies that the *µ* values may be predicted from the initial glyphosate concentrations. The Levenspiel model was the second-best model to explain the experimental data, with *F*, *P*, and *R* ^*2*^ values of 11.69, 0.018, and 0.873, respectively. The Tseng and Wayman model was poorly consistent with the observed kinetic data with *F*, *P*, and *R* ^*2*^ values of 2.59, 0.229, and 0.693, respectively. By contrast, the Yano and Koga and Webb models did not fit the observed *µ* values at all. In fact, these two models exhibited very high *P* values and *R* ^*2*^ equals 0, which means that no variation in the parameter *µ* can be related to that of the initial glyphosate concentration when using these two models. The values of the kinetic parameters of the models are depicted in Table 2. The *µ*_*max*_, *K*_*s*_, and *K*_*i*_ were determined to be 0.94 1/Day, 1.33 mg/L, and 9.97 mg/L, respectively, for the Haldane-Andrews model. The low value of *K*_*s*_ suggests a high affinity between glyphosate and the consortium RH1 enzymes implicated in its breakdown. On the other hand, the value of *K*_*i*_, which refers to as a specific concentration of glyphosate for which *µ* equals *µ*_*max*_/2 when *µ* decreases with increasing glyphosate concentration, is relatively low, suggesting that the bacterial system used demonstrated low tolerance to elevated glyphosate concentrations.

**TABLE 1:** Estimation of the statistical parameters (coefficient of correlation (*R* ^*2*^), *F* and *P* values) for the kinetic models evaluated for the fitting of experimental data on cell growth using glyphosate as a growth substrate.

Model	F value	P value^*^	R^2^
Haldane-Andrews	65.49	0.00074	0.976
Yano and Koga	0.36	0.819	0
Tseng and Wayman	2.59	0.229	0.693
Webb	0.95	0.538	0
Levenspiel	11.69	0.018	0.873

^*^ *P value* < 0.05 is considered to be significant.

**TABLE 2:** Estimated values of kinetic parameters for the five kinetic models tested for the microbial consortium RH1 growth.

Model	Value of estimated kinetic parameters						
	µ_max_ (1/Day)	K_s_ (mg/L)	K_i_ (mg/L)	K_1_ (mg/L)	K_2_ (mg/L)	S_m_ (mg/L)	β
Haldane-Andrews	0.94	1.33	9.97	-	-	-	-
Yano and Koga	1.79	55.27	-	0.35	0.13	-	-
Tseng and Wayman	0.034	8.54	0.0011	-	-	173.94	-
Webb	0.074	429.88	0.16	-	-	-	1.74
Levenspiel	0.32	0.18	186.4	-	-	-	-

On the other hand, the parameters that enabled the calculation of *S*_*min*_, along with the value of the latter, are presented in Table 3. *S*_*min*_ refers to the minimum concentration of the pollutant at which it is used as a growth substrate. That is to say that as long as the concentration of the pollutant (*S*) is greater than *S*_*min*_, the pollutant is metabolically degraded, which means it is driven into primary metabolism, serving as a growth substrate. However, if *S* < *S*_*min*_, the contaminant can no longer sustain microbial growth; instead, it is cometabolically biotransformed (Salah-Tazdaït and Tazdaït, 2024).

**TABLE 3:** Kinetic parameters of glyphosate degradation by the bacterial consortium RH1.

Kinetic parameter	Value	R^2^
b (First-order decay coefficient (1/Day)	0.18	0.94
K_s_ (mg/L)	66.66	0.96
q_max_ (1/Day)	0.017	0.96
S_min_ (mg/L)	0.29	―

The kinetic parameters for glyphosate biodegradation were determined as follows: *q*_*max*_ at 0.017 1/Day, and *K*_*s*_ at 66.66 mg/L, with high *R* ^*2*^ values. These results confirmed that the consortium culture can decompose glyphosate efficiently and can be successfully applied for the bioremediation of sites contaminated with this herbicide.

On the other hand, the low value of *S*_*min*_ (0.29 mg/L) of glyphosate, which is inferior to the glyphosate concentration range tested (1–200 mg/L), indicates that the bacterial consortium used the pesticide as a growth substrate, transforming it into simple and harmless mineral compounds through serving as carbon source and possibly also as nitrogen or even phosphorous source, which makes the mixed culture used a good candidate for efficiently decontaminating liquid and solid media polluted with low glyphosate levels (<0.29 mg/L).

#### 3.6.2 Balanced chemical reaction for the biodegradation of glyphosate using the bacterial consortium RH1

From the standpoint of environmental bioremediation, when a contaminant enters environmental media, it may undergo several biotic or abiotic chemical reactions, including hydrolysis, oxidation, or reduction, all of which are governed by stoichiometric principles. Consequently, the application of the stoichiometric method facilitates the prediction of the quantity of pollutant involved within a biological or chemical system, its degradation over time, and the expected yield of each product. The predicted balanced chemical reaction for the biodegradation of glyphosate by the bacterial consortium utilized in this work is presented below:

$$\begin{array}{c} C_{3}H_{8}{\text{NO}}_{5}P+2{\text{KNO}}_{3}+0.8O_{2} \end{array}\;\to \;\begin{array}{c} 0.2C_{5}H_{7}{\text{NO}}_{2}+1.4N_{2}O+2{\text{CO}}_{2}+2.8H_{2}O+{\text{KH}}_{2}{\text{PO}}_{4} \end{array}$$

This reaction yields a cell mass represented by an empirical chemical formula that includes only the primary elements (carbon, nitrogen, oxygen, and hydrogen), which suffices for most practical uses. The same reaction indicates that the aerobic degradation of 1 kg of glyphosate through mineralization necessitates 0.15 kg of O_2_, 1.19 kg of KNO_3_, and yields 0.13 kg of cells, 0.36 kg of N_2_O, 0.52 kg of CO_2_, 0.29 kg of H_2_O, and 0.8 kg of KH_2_PO_4_. The mass balance determined here estimates the amount of the nutrient KNO_3_ necessary to enhance the growth and breakdown capabilities of the consortium RH1 engaged in the detoxification of glyphosate. It could be applied in a real bioremediation configuration when dealing with glyphosate-contaminated sites.

## 4 Discussion

The bioremediation of contaminated sites by pesticides has been the subject of numerous studies focusing on single microbial strains. However, research has shown that their effectiveness is low in a complex ecosystem, which has led to a shift in research towards the use of active microorganisms to enhance the biodegradation of these pollutants: the consortium.

In this study, four actinobacterial strains are used to form the RH1 consortium. These strains have previously been identified as (*Streptomyces* sp. strain SPA2 (accession number pp413753), *Streptomyces rochei*. strain IT (accession number pp413751), *Streptomyces variabilis*. strain Herb (accession number pp413750), and *Streptomyces griseoincarnatus*. strain SC (accession number PP413754) and confirmed its activity in individually degrading glyphosate as the sole source of carbon at a concentration of 50 mg/L under optimal conditions: a temperature of 30 °C, pH 7.2, and an inoculum size of 4% (Rebai et al., 2025).

Assessing the antagonism between the bacteria used in the consortium is very important for studying the interactions between these bacteria through the secretion of secondary metabolites that can inhibit or limit the growth of a strain, and their metabolic activity, which allows the creation of a balanced, stable and effective consortium to degrade pesticides (Che and Men, 2019). The antagonism test of strains Herb, IT, SC, and SPA2 showed a negative result, which explains that these actinobacterial strains could be cultured together to form the consortium RH1. This result is in agreement with various studies that have shown an absence of antagonisms between different microorganisms, such as the study by Saez et al., 2018 who tested the antagonistic activity between the fungus *Trametes versicolor* S5NG1 and the actinobacteria *Streptomyces* sp. A2, A5, A11, and M7. Antezana et al. (2022) studied the antagonistic interactions between five actinobacteria (*Streptomyces* sp. A5, M7, MC1, *Amycolatopsis tucumanensis* DSM 45259T, and *Micromonospora* sp. A10).

The growth of bacterial consortium and its ability to degrade were tested at different concentrations of glyphosate as the sole source of carbon, to determine the effect of glyphosate concentration on the consortium RH1.

The growth of the actinobacterial consortium in the liquid medium MSM showed an inverse correlation, with dry mass decreasing as glyphosate concentrations increased. This explains the toxicity of high concentrations of glyphosate on the actinobacterial consortium by inhibiting the key enzymes involved in their degradation. Similar results have been obtained in many previous studies. Manogaran et al. (2018) reported that high concentrations of glyphosate (150 ppm–400 ppm) have an inhibitory effect on the growth of the bacterium *Burkholderia vietnamiensis* strain AQ5-12. The study of Tazdaït et al. (2018) found that high concentrations of glyphosate (2 and 5 g/L) inhibited the growth of microorganisms in activated sludge.

At a concentration of 50 mg/L, the growth of the consortium reached 1.07 mg/mL by day 12, which is higher than that of the single strains Herb, IT, SC, and SPA2 (Rebai et al., 2025). The metabolic complementary action among the actinobacteria of the consortium RH1 explains this result. The same results were found in the study by Fuentes et al. (2013), who observed that the growth of the actinobacterium consortium is higher than in cultures in the presence of a mixture of dibasic chlorophenol and chlorpyrifos.

The microbial consortium’s ability to degrade glyphosate at different concentrations as the sole source of carbon was evaluated by a colorimetric method proposed by Bhaskara and Nagaraja. (2006). This method is simple and fast, allowing for the quantification of glyphosate by a spectrophotometric method based on a chemical reaction between glyphosate and ninhydrin in the presence of a catalyst, molybdate. This reaction results in the formation of a colored product (Ruhemann’s purple) detected at the wavelength 570 nm (Xu et al., 2018).

The obtained results showed that the consortium RH1 was able to remove the glyphosate at all tested concentrations, but with different efficiency depending on the concentration of glyphosate, as the degradation rate decreased at higher concentrations (100 mg/L and 200 mg/L). This result confirms that low concentrations of glyphosate are less toxic and allow bacteria to break down the glyphosate substrate, and that as concentrations increase, the inhibitory effect on bacteria increases, which affects the enzymes involved in their degradation. This inhibitory effect of high concentrations has been reported in various studies (Manogaran et al., 2018; Tazdaït et al., 2018).

The consortium RH1 degraded 92.06% of the initial glyphosate concentration (50 mg/L) within 15 days under optimal conditions (Temperature 30 °C, pH 7.2, and inoculum volume 4%). This rate of biodegradation is maximal compared to the single strains Herb, IT, SC, and SPA2, which degraded 78.36%, 48.82%, 73.56%, and 68.22% respectively, at the same conditions (Rebai et al., 2025). This result confirms the effectiveness of the consortium compared to single strains due to the variation in metabolic pathways in glyphosate degradation, and synergistic interaction between strains of the consortium (Smith et al., 2005; Martínez et al., 2008). Similar results were found by different researchers. The study by Pang et al. (2023) demonstrated the highest efficiency of a new microbial consortium, MF0904, which was able to degrade 100% of the initial 25 mg/L methomyl concentration over 96 h. Książek-Trela et al. (2025) found the same results with the consortium consisting of four bacterial strains: *Pseudomonas* sp. 10Kp8 - A1, *Pseudomonas chlororaphis* subsp. *aureofaciens* strain B19 - A2, *Pseudomonas baetica* strain JZY4-9 - C1, and *Streptomyces atratus* strain ROA017 - D1, which exhibited the highest rate of biodegradation of the herbicide diflufenican.

There are a few reports on the biodegradation of glyphosate by bacterial consortia, such as the study of Malla et al. (2023), who studied the biodegradation of three pesticides—chlorpyrifos, cypermethrin, and glyphosate by the C3 consortium consisting of three bacteria: *Lactobacillus plantarum*, *Lactobacillus rhamnosus*, and *Bacillus shackletonii*. Their study revealed that the C3 consortium had degraded 87.01% of the glyphosate during a 15-day incubation period. The work of Góngora-Echeverría et al. (2020) on the degradation of glyphosate and other pesticides (atrazine, carbofuran) by a microbial consortium showed that the microbial consortium degraded more than 90% of the glyphosate. Zhang et al. (2024) studied the ability of the consortium YS622 to degrade glyphosate, and they found that it was very effective in degrading 100% of 50 mg/L glyphosate within 36 h. The results in this study demonstrate the bioremediation capacity of pesticides by actinobacteria consortium, which is confirmed by different studies who studied the bioremediation of different pollutants (Antezana et al., 2022; Fuentes et al., 2017; Fuentes et al., 2011). This ability of actinobacteria is explained by their richness in secondary metabolism, such as enzymes involved in biodegradation, as well as the production of products such as siderophores and extracellular polymer substances, which have an important role in the immobilization of organic contaminants and the immobilization of heavy metals, In addition, they can resist extreme environmental conditions (Makarani and Kaushal, 2025).

The total organic carbon test indicates a remarkable and higher decrease (91.03%) of initial concentration 50 mg/L compared to single strains Herb, IT, SC, and SPA2 which showed a reduction rate of 82.06%, 47.96%, 67.12%, 56.11%, and respectively (Rebai et al., 2025), which indicates that the consortium RH1 is more effective in the mineralization of the pesticide.

The ATR-FTIR analysis demonstrates a modest variation in peaks between the consortium RH1 and the control sample, which may indicate a structural alteration, suggesting possible biodegradation of the herbicide glyphosate.

It has been documented in literature that numerous microbial strains, including bacteria and fungi, have the capability to break down pesticides. For instance, in a recent study, a strain of cyanobacteria (*Nostoc* sp. PCC7120) was found to tolerate the neurotoxic insecticide thiamethoxam at a concentration of 10 mg/L. This concentration exhibited a growth-stimulating effect, with a *µ* value of about 0.37 1/Day (Zhu et al., 2025). With respect to the use of microorganisms for bioremediation, it is necessary to perform investigations to assess the limitations of the microbial system in regard to the particular contaminant elimination, and it is also essential to identify the microbial inhibitory growth that may occur in response to an increase in the concentration of the contaminant. Results on the effect of the initial glyphosate concentration on consortium RH1 growth show that for initial glyphosate concentrations below 10 mg/L, the *µ* value increases with the increase in the initial concentration of glyphosate. When the glyphosate concentration exceeds 10 mg/L, the growth of the microbial consortium slows down due to the inhibitory effect caused by high glyphosate concentrations. This phenomenon, called the hormesis effect, is referred to as a dose-response relationship exhibiting a stimulatory impact at low concentrations and an inhibitory effect at high concentrations. This effect is prevalent when dealing with the bioremediation of xenobiotics and could improve the process of bioremediation through precise knowledge of tolerance thresholds for contaminants in microorganisms (Agathokleous et al., 2023). The inhibition of the consortium RH1 growth observed in this study could be due to the inhibition by glyphosate and/or its metabolites of specific enzymes of the primary metabolism responsible for energy and reducing power generation.

The concentration of xenobiotics above which the growth inhibition occurs depends on their chemical structures and the physiological and biochemical capabilities of the degrading microorganisms used in both axenic and consortium systems. For example, in the study by Lin et al. (2026), the growth of the bacterium *Pandoraea pnomenusa* ZQ05 was inhibited by concentrations of acephate greater than 200 mg/L. Feng et al. (2025) reported that the proliferation of the actinobacterial strain *Streptomyces* sp. FW-32 was stopped entirely by 3,6-dibromocarbazole tested at 10 mg/L within 3 days of incubation. In another study, initial concentrations of acetochlor greater than 20 mg/L significantly inhibited the growth of *Klebsiella michiganensis* ES15. Additionally, acidic and alkaline conditions hindered the proliferation of the strain, even at the optimal acetochlor concentration for growth (20 mg/L) (Zhao et al., 2025). Zhang et al. (2024) suggested that the growth of a microbial consortium YS622 (dominated mainly by *Ochrobactrum*, *Cloacibacterium*, and *Azospirillum*) was inhibited by initial concentrations of glyphosate superior to 59 mg/L.

The *R* ^*2*^ obtained with the Haldane-Andrews model indicates substantial model reliability as it surpasses the critical value of 0.8, below which the experimental data no longer fit the predicted ones (Sonwani et al., 2019). Reports on the use of kinetic models to explain the microbial inhibitory growth induced by xenobiotics, especially glyphosate, used as substrates, are scarce. Mishra et al. (2024) tested *Stenotrophomonas maltophilia* (HE963840.1) for the biodegradation of methyl orange dye and found the Haldane-Andrews model was a very good fit for the growth kinetics data of the bacterium tested, with *R* ^*2*^ equaling 0.99. The authors reported *K*_*s*_ and *K*_*i*_ values of 78 mg/L and 156 mg/L, respectively. Another study reported that the Edwards and Haldane-Andrews models were the most appropriate for describing the breakdown of chlorpyrifos by *Pseudomonas aeruginosa* and *Methylobacterium zatmanii* (*K*_*s*_ = 3.9 mg/L, *K*_*i*_ = 76 mg/L) and dichlorvos by *Pseudomonas aeruginosa* and *Taonella mepensis* (*K*_*s*_ = 52 mg/L, *K*_*i*_ = 1,279 mg/L), respectively (Gaonkar et al., 2019). Lin et al. (2026) who investigated the biodegradation of acephate by the bacterium *Pandoraea pnomenusa* found that the Andrews equation provided a good fit to the biodegradation kinetics data, with *K*_*s*_ = 112017.6 mg/L and *K*_*i*_ = 0.04 mg/L. The Yano and Koga model exhibited the best fit for explaining the biodegradation of glyphosate (*K*_*s*_ = 64 mg/L, *K*_*1*_ = 2,400 mg/L, *K*_*2*_ = 1,230 mg/L) (Tazdaït et al., 2018), and malathion (*K*_*s*_ = 87,806 mg/L, *K*_*1*_ = 6.25 mg/L, *K*_*2*_ = 3.6 mg/L) (Tazdaït et al., 2013) by a mixed culture of activated sludge, while the model of Haldane-Andrews was the best fitted for glyphosate removal by a mixed culture of *Ochrobactrum*, *Cloacibacterium*, and *Azospirillum* (Zhang et al., 2024). Rajamanickam et al. (2017) examined the capability of a microbial consortium consisting mainly of *Escherichia coli*, *Bacillus* species and *Pseudomonas* species to break down toluene in batch experiments. It was demonstrated that the Levenspiel model accurately represented the experimental data (*R* ^*2*^ = 0.98) and reported a *K*_*s*_ of 20 mg/L and *K*_*i*_ of 230 mg/L.

The estimated kinetic parameters *q*_*max*_ (0.017 1/Day) and *K*_*s*_ (66.66 mg/L) can serve to assess the biodegradation performance of consortium RH1 and act as input parameters for glyphosate removal in real-scale treatment processes, such as biostimulation or bioaugmentation. The half-rate constant *K*_*s*_ determined in this study, which designates the concentration of glyphosate for which *q* equals *q*_*max*_/2 and whose reverse value represents the affinity of the consortium RH1 for glyphosate, is relatively low compared to that determined for the degradation of glyphosate by a bacterial consortium (*Ochrobactrum*, *Cloacibacterium*, and *Azospirillum*) (139 mg/L) (Zhang et al., 2024), but similar to that reported using activated sludge culture (64 mg/L) for the same pesticide (Tazdaït et al., 2018). The low *K*_*s*_ value found in this study shows a relatively high affinity between the microbial system used and glyphosate, which can be a positive addition to the removal of glyphosate by the consortium RH1 in real-time wastewater settings. The results demonstrated effective pesticide degradation by the consortium. Furthermore, the consortium may have potential applications in the bioremediation of contaminated soils, although additional studies are required.

Glyphosate biodegradation by microbial consortia generally proceeds through two major metabolic pathways: the sarcosine pathway mediated by the C–P lyase system and the AMPA pathway initiated by glyphosate oxidoreductase (Badani et al., 2024; Morales-Olivares et al., 2025). In the C–P lyase route, cleavage of the carbon–phosphorus bond produces sarcosine and inorganic phosphate, followed by conversion of sarcosine into glycine and formaldehyde through sarcosine oxidase activity (soxA). In the AMPA pathway, glyphosate oxidoreductase encoded by the dadA gene converts glyphosate into aminomethylphosphonic acid (AMPA) and glyoxylate (Morales et al., 2020; Mulati et al., 2025), after which AMPA is further mineralized through the phn-encoded C–P lyase complex. Genes associated with these pathways, particularly the phn operon (phnC–phnP), phnJ, dadA, and soxA, have been widely reported in glyphosate-degrading bacteria (Mulati et al., 2025) and are considered essential for phosphonate transport and C–P bond cleavage. The high degradation efficiency and TOC removal observed in the RH1 consortium suggest that complementary metabolic interactions among the *Streptomyces* strains may enable efficient glyphosate mineralization through combined AMPA and sarcosine pathways. Such synergistic activity could explain the superior biodegradation performance of the consortium compared with individual strains. Although functional genes were not directly characterized in this study, the observed biodegradation behavior strongly indicates the involvement of conserved phosphonate-degrading genetic systems, warranting future molecular analyses targeting genes such as phnJ, dadA, and soxA.

## 5 Conclusion

This study was based on the test of the capacity of the RH1 consortium to degrade glyphosate at different concentrations of glyphosate as the only carbon source at optimal conditions (temperature of 30 °C, pH 7.2 and inoculum size 4%), to determine the inhibitory effect of concentration on the activity of bacteria and, as a consequence, on the biodegradation of glyphosate. The consortium was made up of four actinobacterial strains previously studied, and they confirmed their ability to degrade the same herbicide. The results obtained indicated that the consortium was capable of growing and degrading glyphosate at lower concentrations (from 1 mg/L to 50 mg/L), but its efficacy was low at high concentrations (100 mg/L and 200 mg/L). The effectiveness of the consortium compared to individual bacteria was tested at a concentration of 50 mg/L of glyphosate. The results showed that RH1 was more effective than single strains, with maximum degradation rates (92.06%), as well as the rate of TOC (91.03%). The ATR FTR results showed a variation in the chemical structure of glyphosate after biodegradation, after incubation with the consortium. The growth kinetic study showed that the Haldane-Andrews model fit the experimental data well. It was noteworthy that the calculated *S*_*min*_, which is low, suggests the possibility of applying the constructed consortium to the degradation of glyphosate, through mineralization, in real environmental conditions. The findings of the present study demonstrate the actinomycete consortium RH1’s ability to be used in contaminated environments as a bioremediation agent, increasing our understanding of actinomycete species tolerance for treating xenobiotics. This provides the underpinning for enhancing microbial bioremediation systems for glyphosate and other herbicide contamination. However, the bioremediation potential of the consortium RH1 should be assessed in relation to the influence of abiotic parameters, such as the presence of organic and inorganic pollutants, and fluctuating temperature and pH, which can significantly impact the biodegradation potential under real bioremediation conditions. Further validation is required because the present study was limited to controlled laboratory conditions with glyphosate as the sole carbon source and did not include co-contaminants or fluctuating environmental parameters. Future work will test RH1 under *ex situ* and *in situ* field experiments, clarify inhibition at high glyphosate concentrations, and elucidate the metabolic pathways/genetic basis of glyphosate degradation to better support practical bioremediation.

## Data Availability

The original contributions presented in the study are included in the article/supplementary material, further inquiries can be directed to the corresponding author.
